# Supplementary material for: Interval forecasts of weekly incident and cumulative COVID-19 mortality in the United States: A comparison of combining methods
Source: PLoS One. 2022 Mar 29;17(3):e0266096. doi: 10.1371/journal.pone.0266096 (PMC8963571; doi:10.1371/journal.pone.0266096)
Supplement: S1 Table — (PDF) [file pone.0266096.s002.pdf]

S1 Table. Individual forecasting models

Contributors	Short model name	Model description*	Access and licencing information<br>Citations
Wattanachit N, Ray EL, Reich N	COVID hub-ensemble	An ensemble, or model average, of submitted forecasts to the COVID-19 Forecast Hub.	https://github.com/reichlab/covid19-forecast-hub/tree/master/data-processed/COVIDhub-ensemble<br>https://www.medrxiv.org/content/10.1101/2020.08.19.20177493v1
COMPARTMENTAL			
Tomar V, Jain C	Auquan-SEIR [a]	Modified SEIR model with compartments for reported and unreported infections. Non-linear mixed effects curve-fitting.	https://github.com/reichlab/covid19-forecast-hub/tree/master/data-processed/Auquan-SEIR
Panano B.	BPangano-RtDriven	Projects infections and deaths for 223 locations using an SIR model.	https://github.com/reichlab/covid19-forecast-hub/tree/master/data-processed/BPagano-RtDriven<br><br>https://bobpagano.com/covid-19-modeling/
Carlson E, Henderson M, Kelly C, Kofman I, Zhang X	CovidActNow-SEIR_CAN	SEIR model forecasts of cumulative deaths, incident deaths, incident hospitalizations by fitting predicted cases, deaths, and hospitalizations to the observations.	https://github.com/reichlab/covid19-forecast-hub/tree/master/data-processed/CovidActNow-SEIR_CAN
Li ML, Bouardi HT, Lami OS, Trikalinos TA, Trichakis NK, Bertsimas D	CovidAnalytics-DELPHI	SEIR model augmented with underdetection and interventions. Projections account for reopening and assume interventions would be re-enacted if cases continue to climb.	https://github.com/reichlab/covid19-forecast-hub/tree/master/data-processed/CovidAnalytics-DELPHI<br>https://www.covidanalytics.io/DELPHI_documentation_pdf
Chhatwal J, Ayer T, Linas B, Dalgic O, Mueller P, Adee M, Ladd MA, Xiao J	Covid19Sim-Simulator	An interactive tool that uses a validated SEIR compartment model.	https://github.com/reichlab/covid19-forecast-hub/tree/master/data-processed/Covid19Sim-Simulator
Pei S, Yamana T, Kandula S, Yang W, Galanti M, Shaman J	CU-select	Metapopulation county-level SEIR model for projecting future COVID-19 incidence and deaths. This forecast is the scenario we believe to be most plausible given the current setting.	https://github.com/reichlab/covid19-forecast-hub/tree/master/data-processed/CU-select<br>https://doi.org/10.1101/2020.03.21.20040303<br>https://www.medrxiv.org/content/10.1101/2020.05.04.20090670v2
Pei S, Yamana T, Kandula S, Yang W, Galanti M, Shaman J	CU-nochange	This metapopulation county-level SEIR model assumes that current contact rates will	https://github.com/reichlab/covid19-forecast-hub/tree/master/data-processed/CU-nochange

		remain unchanged in the future.	https://doi.org/10.1101/2020.03.21.20040303
Max A, Epshteyn A, Kang B, Li C-L, Sava D, Parish D, Miller D, Kanal E, Liu H, Nakhost H, Jones I, Lai J, Repenning J, Yoon J, Ramasamy K, Zhang L, Le L, Nikoltchev M, Siegler M, Dusenberry M, Yoder N, Rozenfeld O, Rangaswamy P, Sinha R, Xie R, Arik S, Singh S, Tsai T, Pfister T, Menon V, Karande V, Y, Li Y	Google-Harvard-CPF	Our model improves upon standard compartmental models by using temporally and spatially rich data, and integrating covariate encodings into compartment transitions via end-to-end learning.	https://github.com/reichlab/covid19-forecast-hub/tree/master/data-processed/Google_Harvard-CPF https://arxiv.org/abs/2008.00646
Lemaitre JC, Bi Q , Hulse JD, Grabowski MK, Grantz KH, Kaminsky J, Lauer SA, Lee EC, Meredith HR, Perez-Saez J, Truelove SA, Keegan LT, Kaminsky K, Shah S, Wills J, Aquilanti P-Y, Raman K, Subramaniyan A, Thursam G, Tran A.	JHU_IDD-CovidSP	County-level metapopulation model with commuting and stochastic SEIR disease dynamics with social-distancing indicators.	https://github.com/reichlab/covid19-forecast-hub/tree/master/data-processed/JHU_IDD-CovidSP https://doi.org/10.1038/s41598-021-86811-0
Kinsey M, Tallaksen K, Obrecht RF, Asher L, Costello C, Kelbaugh M, Wilson S	JHUAPL_Bucky	Metapopulation model using public mobility data. Local parameters (case reporting rates, doubling times, etc) are estimated using data from CSSE and CDC scenario 5. Primary output is case incidence.	https://github.com/reichlab/covid19-forecast-hub/tree/master/data-processed/JHUAPL-Bucky
Baek J, Farias V, Georgescu A, Levi R, Sinha D, Wilde J, Zheng A	MITCovAlliance-SIR	SIR model trained on public heath regions. SIR parameters are functions of static demographic and time-varying mobility features. Two-stage approach that first learns magnitude of peak infections.	https://github.com/reichlab/covid19-forecast-hub/tree/master/data-processed/MITCovAlliance-SIR https://arxiv.org/abs/2006.06373
Vespignani A, Chinazzi M, Davis JT, Mu K, Pastore y Piontti A, Samay N, Xiong X, Halloran ME, Longini IM, Dean NE, Viboud C, Sun K, Litvinova M,	MOBS-GLEAM_COVID	Metapopulation, age structured SLIR model. Superimposed on the worldwide population and mobility layers is an agent-based epidemic model that defines the infection and population dynamics. Makes predictions about the future	https://github.com/reichlab/covid19-forecast-hub/tree/master/data-processed/MOBS-GLEAM_COVID https://uploads-ssl.webflow.com/58e6558acc00ee8e4536c1f5/5e8bab44f5baae4c1c2a75d2_GLEAM_web.pdf

Gioannini C, Rossi L, Ajelli M		that are dependent on the assumption that current interventions continue.	
Gao Z, Li C, Zheng S, Bian J, Xie X, LiuT-Y	MSRA-DeepST	A deep spatio-temporal network with knowledge based SEIR as a regularier under the assumption of spatio-temporal process in pandemic of different regions.	https://github.com/reichlab/covid19-forecast-hub/tree/master/data-processed/MSRA-DeepST
Espana G, Oidtman R, Cavany S, Costello A, Wieler A, Lerch A, Barbera C, Poterek M, Tran Q, Moore S, Perkins A	NotreDame-Mobility	Ensemble of nine models that are identical except that they are driven by different mobility indices from Apple and Google. The model underlying each is a deterministic, SEIR-like model.	https://github.com/reichlab/covid19-forecast-hub/tree/master/data-processed/NotreDame-mobility
Koyluoglu U, Milliken J	OliverWyman-Navigator	Forecasts and scenario analysis for Detected and Undetected cases and death counts following a compartmental formulation with non-stationary transition rates.	https://github.com/reichlab/covid19-forecast-hub/tree/master/data-processed/OliverWyman-Navigator
Turtle J, Ben-Nun M, Riley P	PSI-DRAFT	A stochastic/deterministic, single-population SEIRX model that stratifies by both age distribution and disease severity and includes generic intervention fitting.	https://github.com/reichlab/covid19-forecast-hub/tree/master/data-processed/PSI-DRAFT
Shi Y, Shah T, Ban X	RPI-UW-Mob_Collision	A mobility-informed simplified SIR model motivated by collision theory.	https://github.com/reichlab/covid19-forecast-hub/tree/master/data-processed/RPI-UW-Mob-Collision https://www.medrxiv.org/content/10.1101/2020.07.25.20162016v1
Snyder TL, Wilson DD	SWC-TerminusCM	Mechanistic compartmental model using disease parameter estimates from literature. It uses Bayesian inference to predict the most likely model parameters.	https://github.com/reichlab/covid19-forecast-hub/tree/master/data-processed/SWC-TerminusCM
Cobey S, Arevalo P, Baskerville E, Carran S, Gostic K, McGough L, Ranjeva S, Wen F	UChicago-COVIDIL	Compartmental, age-structured SEIR model that infers past SARS-CoV-2 transmission rates and forecasts mortality under current and hypothetical public health interventions.	https://github.com/reichlab/covid19-forecast-hub/tree/master/data-processed/UChicago-CovidIL
Gu Q, Xu P, Chen J, Wang L, Zou D, Zhang W	UCLA-SuEIR	Variant of the SEIR model considering both untested and unreported cases. The model considers reopening and assumes susceptible	https://github.com/reichlab/covid19-forecast-hub/tree/master/data-processed/UCLA-SuEIR

		population will increase after the reopen.	https://www.medrxiv.org/content/10.1101/2020.05.24.20111989v1
Chen YQ, Zhao Y, Guo L	UCM-MESALab-FoGSEIR	FoGSEIR model is a modification of integer order SEIR model considering fractional integrals. The model considers the age structure and reopening intervention to minimize infections and deaths.	https://github.com/reichlab/covid19-forecast-hub/tree/master/data-processed/UCM_MESALab-FoGSEIR
Sheldon D, Gibson G, Reich N	UMass-MechBayes	Bayesian compartmental model with observations on cumulative case counts and cumulative deaths. Model is fit independently to each state. Model includes observation noise and a case detection rate.	https://github.com/reichlab/covid19-forecast-hub/tree/master/data-processed/UMass-MechBayes
Mayo ML, Rowland MA, Parno MD, Detwiller ID, Farthing MW, England WP George GE	USACE-ERDC_SEIR	The ERDC SEIR model makes predictions of several variables (e.g., reported new/cumulative cases per day, etc.). Model parameters are estimated using historical data using Bayesian inference.	https://github.com/reichlab/covid19-forecast-hub/tree/master/data-processed/USACE-ERDC_SEIR
Jain S, Tiwari A, Deva A, Kulkarni M, Shingi S, Bannur N, White J, Merugu S, Raval A	Wadhwani_AI-BayesOpt	A novel model-agnostic Bayesian optimization ("BayesOpt") approach for learning the parameters of our SEIR model from observed data.	https://github.com/reichlab/covid19-forecast-hub/tree/master/data-processed/Wadhwani_AI-BayesOpt
Gu Y	YYG-ParamSearch	Based on the SEIR model with hyperparameter optimization to make daily projections regarding COVID-19 infections and deaths in 50 US states. The model accounts for state reopenings and its effects on infections and deaths.	https://github.com/reichlab/covid19-forecast-hub/tree/master/data-processed/YYG-ParamSearch https://covid19-projections.com/about/
NON-COMPARTMENTAL			
O'Dea E	CEID-Walk	A random walk model with drift. A least squares line is fitted to the tail observations of a target time series to estimate the drift and step variance of a random walk model.	https://github.com/reichlab/covid19-forecast-hub/blob/master/data-processed/CEID-Walk/metadata-CEID-Walk.txt
Green A, Hu A, Jahja M, Ventura V, Wasserman L, Tibshirani Rob, Shankar V, Bien J, Brooks L,	CMU-Timeseries [b]	A basic AR-type time series model fit using case counts and deaths as features.	https://github.com/reichlab/covid19-forecast-hub/tree/master/data-processed/CMU-TimeSeries

Narasimhan B, Rajanala S, Rumack A, Simon N, Sharpnack J, McDonald D(University of British Columbia), Ryan Tibshirani (Senior author, and the Delphi COVID-19 Response Team			
Wang Y, Zeng D, Wang Q, Xie S	Columbia_UNC-SurvCon	Survival-convolution model with piece-wise transmission rates that incorporates latent incubation period and provides time-varying effective reproductive number.	https://github.com/reichlab/covid19-forecast-hub/tree/master/data-processed/Columbia_UNC-SurvCon https://www.frontiersin.org/article/10.3389/fpubh.2020.00325
Ray EL, Tibshirani R	COVIDhub-baseline	Baseline prediction model.	https://github.com/reichlab/covid19-forecast-hub/tree/master/data-processed/COVIDhub-baseline
Kalantari R, Zhou M.	DDS-NBDS	Jointly modeling daily deaths and cases using a negative binomial distribution based nonparametric Bayesian generalized linear dynamical system.	https://github.com/reichlab/covid19-forecast-hub/tree/master/data-processed/DDS-NBDS https://dds-covid19.github.io/
Sherratt K, Bosse N, Abbott S, Hellewell J, Meakin S, Munday J, Funk S	epiforecasts-ensemble1	A deaths forecast using the renewal equation and time-series forecasts of the time-varying reproduction number.	https://github.com/reichlab/covid19-forecast-hub/tree/master/data-processed/epiforecasts-ensemble1 https://doi.org/10.12688/wellcomeopenres.16006.1
Keskinocak P, Aglar BEO, Baxter A, Asplund J, Serban N	GT_CHHS-COVID19	Agent-based simulation model to project COVID19 infection spread.	https://github.com/reichlab/covid19-forecast-hub/tree/master/data-processed/GT_CHHS-COVID19
Prakash BA, Rodriguez A, Cui J, Tabassum A, Adhikari B, Sun J, Xiao D, Qiang C	GT-DeepCOVID	Data-driven approach based on deep learning for forecasting mortality and hospitalizations using syndromic, clinical, demographic, mobility and point-of-care data.	https://github.com/reichlab/covid19-forecast-hub/tree/master/data-processed/GT-DeepCOVID
Murry C and the IHME-CurveFitTeam	IHME-CurveFit	Non-linear mixed effects curve-fitting. This model makes predictions about the future that are dependent on the assumption that current interventions continue.	https://github.com/reichlab/covid19-forecast-hub/tree/master/data-processed/IHME-CurveFit https://www.medrxiv.org/content/10.1101/2020.03.27.20043752v1

Wang L, Wang G, Gao L, Li X, Yu S, Kim M, Wang Y, Gu Z.	IowaStateLW-STEM	A nonparametric space-time disease transmission model. The projections assume that the data used is reliable, the future will continue to follow the current pattern, and current interventions will remain the same till the end of forecasting period.	https://github.com/reichlab/covid19-forecast-hub/tree/master/data-processed/IowaStateLW-STEM https://arxiv.org/abs/2004.14103
Chiang W-H, Mohler G	IUPUI-HkPrMobiDyR	Hawkes processes with Dynamic reproduce number.	https://github.com/reichlab/covid19-forecast-hub/tree/master/data-processed/IUPUI-HkPrMobiDyR<br><br>https://doi.org/10.1101/2020.06.06.20124149
Marshall M, Gardner L, Drew C, Burman E, Nixon K	JHU_CSSE-DECOM	County-level, empirical machine learning model driven by epidemiological, mobility, demographic, and behavioral data.	https://github.com/reichlab/covid19-forecast-hub/tree/master/data-processed/JHU_CSSE-DECOM
Karlem D	Karlen-pypm	Discrete-time difference equations with long periods of constant transmission rate	https://github.com/reichlab/covid19-forecast-hub/tree/master/data-processed/Karlen-pypm<br><br>https://arxiv.org/abs/2007.07156
Osthus D, Del Valle S, Manore C, Weaver B, Castro L, Shelley S, Smith M, Spencer J, Fairchild G, Travis Pitts T, Gerts D, Dauelsberg L, Daughton A, Gorris M, Hornbein B, Israel D, Parikh N, Shutt D, Ziemann A	LANL-GrowthRate	Statistical dynamical growth model accounting for population susceptibility. Makes predictions about the future, unconditional on particular intervention strategies.	https://github.com/reichlab/covid19-forecast-hub/tree/master/data-processed/LANL-GrowthRate
Gao Z, Li C, Cao W, Zheng S, Bian J, Xie X, Liu TY, Zhang S, Ferres JL	Microsoft-DeepSTIA [a]	A deep spatio-temporal network with intervention and hospital gate under the assumption of spatio-temporal process in pandemic of different regions.	https://github.com/reichlab/covid19-forecast-hub/tree/master/data-processed/Microsoft-DeepSTIA
Espana G, Oidtman R, Cavany S, Costello A, Wieler A, Lerch A, Barbera C, Poterek M, Tran Q, Moore S, Perkins A	NotreDame-FRED	Agent-based model developed for influenza with parameters modified to represent the natural history of COVID-19	https://github.com/reichlab/covid19-forecast-hub/tree/master/data-processed/NotreDame-FRED
Walraven R	RobertWalraven-ESG	Multiple skewed gaussian distribution peaks fitted to raw data.	https://github.com/reichlab/covid19-forecast-hub/tree/master/data-processed/RobertWalraven-ESG

Nagraj VP, Turner SD, Hulme-Lowe C	SigSci_TS	Time series forecasting using ARIMA for case forecasts and lagged cases for death forecasts.	https://github.com/reichlab/covid19-forecast-hub/tree/master/data-processed/SigSci-TS
McConnell S, Donaldson B	SteveMcConnell_COVIDComplete	A near-term fatality prediction model that calculates and uses fatality trends at the national and state level, trends in positive virus tests and total virus tests, and age-related demographics for state forecasts. Model forecasts are based on predicting near-term deaths from recent positive virus tests.	https://github.com/reichlab/covid19-forecast-hub/tree/master/data-processed/SteveMcConnell-CovidComplete https://stevemcconnell.com/covid
Bieggel H, Lega J	UA-EpiCovDA	SIR mechanistic model with data assimilation. EpiCovDA is an extension of the EpiGro model. Model parameters are fit to Covid-19 data using a variational data assimilation method. A prior distribution of the parameters is estimated by fitting an SIR Incidence-Cumulative Cases curve to data from states that had at least 1000 cases by 04/01/2020.	https://github.com/reichlab/covid19-forecast-hub/tree/master/data-processed/UA-EpiCovDA
Jin X, Wang Y-X, Yan X	UCSB-ACTS	This data-driven machine learning model makes predictions by referring to other regions with similar growth patterns and assuming the similar development will take place in the current region.	https://github.com/reichlab/covid19-forecast-hub/tree/master/data-processed/UCSB-ACTS
Wu D, Gao L, M Yian, Yu R, Vespignani A, Chinazzi M, Davis JT, Mu K, Pastore y Piontti A, Xiong X	UCSD-NEU_DeepGLEAM	Combines the signal of a discrete stochastic epidemic computational model GLEAM with a deep learning spatiotemporal forecasting framework to further improve predictions.'	https://github.com/reichlab/covid19-forecast-hub/tree/master/data-processed/UCSD_NEU-DeepGLEAM
Corsetti S, Schwarz T	UMich-RidgeTfReg	Nation-level model of confirmed cases and deaths based on ridge regression. No assumptions made about social distancing.	https://github.com/reichlab/covid19-forecast-hub/tree/master/data-processed/UMich-RidgeTfReg
Zhang-James Y, Hess J, Chen S, Wang D, Morley CP, Faraone SV.	UpstateSU_GRU [b]	County-level forecast using recurrent neural network seq2seq model with the Gated recurrent units (GRU)	https://github.com/reichlab/covid19-forecast-hub/tree/master/data-processed/UpstateSU-GRU
Srivastava A, Prasanna VK, Xu FT	USC-SI_kJalpha [b]	A heterogeneous infection rate model with human	https://github.com/reichlab/covid19-forecast-

		mobility for epidemic modeling. Our model adapts to changing trends and provide predictions of confirmed cases and deaths.	hub/tree/master/data-processed/USC-SI_kJalpha<br><br>https://arxiv.org/abs/2007.05180
Srivastava A, Prasanna VK, Xu FT	USC-SI_kJalpha_RF	A heterogeneous infection rate model with human mobility for epidemic modeling. Our model adapts to changing trends and provide predictions of confirmed cases and deaths. We build a random forest, based on the output of USC_SIkJalpha model along with the data on the cumulative case/death, weekly increase, and previous increase. We then sample trees to generate quantile forecasts	https://github.com/reichlab/covid19-forecast-hub/tree/master/data-processed/USC-SI_kJalpha_RF<br><br>https://arxiv.org/abs/2007.05180
Woody S, et al. at the University of Texas	UT-Mobility	This model makes predictions assuming that social distancing patterns, as measured by anonymized mobile-phone GPS traces, remain constant in the future. Only models *first-wave deaths*.	https://github.com/reichlab/covid19-forecast-hub/tree/master/data-processed/UT-Mobility
Mehrotra P, Ivan JI, and the Walmart Labs COVID-19 Team	WalmartLabsML_LogForecasting [a]	A logistic growth prophet forecasting model fit using case counts and deaths as features.The Model is built by Prophet model with logistic growths to forecast the US cumulative deaths. By sampling from uniform distribution to get the quantiles.	https://github.com/reichlab/covid19-forecast-hub/tree/master/data-processed/WalmartLabsML-LogForecasting

* Based on information recorded on the COVID19 Hub with citations as recorded on 18/5/21; [a] Only provided forecasts of numbers of cumulative COVID-19 deaths; [b] Only provided forecasts of numbers of incident COVID-19 deaths.
