# Supplementary material for: Interval forecasts of weekly incident and cumulative COVID-19 mortality in the United States: A comparison of combining methods
Source: PLoS One. 2022 Mar 29;17(3):e0266096. doi: 10.1371/journal.pone.0266096 (PMC8963571; doi:10.1371/journal.pone.0266096)
Supplement: S2 Table — Lower values are better. a best method for each horizon in each column; b score is significantly lower than the mean combination; c score is significantly lower than the median combination. (PDF) [file pone.0266096.s003.pdf]

S2 Table. For incident mortality, 95% interval MIS and MWIS for each prediction horizon.

Method	95% interval MIS					MWIS				
	All	U.S.	High	Med	Low	All	U.S.	High	Med	Low
1 week ahead										
Mean	650	7216	1032	434	98	42.3	677.4	58.9	23.6	7.2
Median	633	8477	908	429	99	40.9	684.3	55.0	22.7 [b]	7.2
Ensemble	633	8931	891	424	97	41.1	704.0	54.5	22.6 [ab]	7.1 [a]
Sym trim	628	7572	951	427	97	41.6 [b]	676.1	57.5	22.7 [b]	7.1 [a]
Exterior trim	663	7756	1030	441	101	43.3	693.8	60.9	23.6	7.3
Interior trim	614	7286	952	405 [ab]	91 [ab]	42.5	684.3	59.3	23.2 [b]	7.2
Envelope	2249	20811	4746	654	254	140.0	2057.9	228.2	60.2	18.8
Inv score	595 [b]	7169 [a]	891 [b]	414 [b]	92 [bc]	40.7 [b]	656.1 [b]	55.9 [b]	23.1 [b]	7.1 [ab]
Inv score tuning	577 [a]	7285	822 [a]	419	94	39.5 [ab]	625.0 [a]	53.5 [ab]	23.5	7.1 [a]
Previous best	719	10217	996	481	121	46.4	715.8	63.9	27.8	8.2
2 weeks ahead										
Mean	744	8237	1217	469	106	54.6	813.5	84.0	27.3	8.0
Median	656	8355	946	467	102	47.1	814.4	62.3	25.9 [b]	7.8 [ab]
Ensemble	684	10151	936	457	102	47.7	854.3	62.1	25.8 [ab]	7.8 [ab]
Sym trim	716	8383	1132	463	102	53.6	809.8	82.3	26.2 [b]	7.9 [b]
Exterior trim	768	8945	1237	477	108	77.9	830.9	154.0	27.3	8.1
Interior trim	750	8209	1267	443	102	58.6	816.8	96.5	26.8 [b]	8.0
Envelope	3220	27179	7049	828	372	249.4	2656.4	508.1	75.5	23.0
Inv score	649 [b]	7586	989 [b]	452 [a]	97 [ab]	53.0	763.2 [b]	82.5	26.7 [b]	7.9 [b]
Inv score tuning	606 [ab]	7479 [a]	845 [a]	465	102	45.7 [a]	727.8 [a]	61.6 [a]	27.4	8.0
Previous best	794	10706	1092	570	138	55.6	906.9	74.3	33.1	9.5
3 weeks ahead										
Mean	801	8813 [a]	1344	477	109	58.5	941.5	85.2	29.7	8.6
Median	731	9992	1046	498	103	54.1	965.5	71.3	29.2	8.3 [a]
Ensemble	727	10531	1016	487	102	53.4	947.9	70.3	29.1 [a]	8.3 [a]
Sym trim	795	9603	1270	487	110	58.4	966.0	84.0	29.4	8.4
Exterior trim	857	10455	1401	497	107	73.1	969.2	128.2	30.0	8.5
Interior trim	780	8910	1285	469	107	61.0	957.2	92.4	29.4	8.6
Envelope	3749	46863	6995	1062	655	251.0	3460.1	446.6	87.8	29.8
Inv score	707 [b]	8974	1079 [b]	460 [a]	96 [a]	56.2 [b]	881.2 [b]	82.3	29.3	8.3 [ab]
Inv score tuning	664 [a]	9009	912 [a]	480	107	52.4 [a]	880.4 [a]	69.6 [a]	30.3	8.7
Previous best	885	11273	1242	658	145	65.7	1121.8	86.0	38.1	10.9
4 weeks ahead										
Mean	924	12738	1403	510	163	66.6	1156.6	92.2	33.6	10.0
Median	873	11671	1302	560	122	64.1	1193.6	83.9	32.7 [a]	9.2 [a]
Ensemble	864	11602	1283	558	120 [ac]	63.9	1192.9	83.3	32.8	9.2 [a]
Sym trim	917	12301	1396	550	134	66.9	1199.0	91.5	33.1	9.3
Exterior trim	1009	14588	1539	546	145	74.4	1204.8	112.9	34.2	9.7
Interior trim	925	12765	1411	508	158	68.3	1173.2	96.5	33.4	10.0
Envelope	6135	128559	8219	1643	1342	295.7	4984.5	452.3	115.6	43.5
Inv score	809 [b]	12128	1161 [b]	481 [abc]	121	63.2 [b]	1073.1 [a]	87.8 [b]	32.7 [ab]	9.5
Inv score tuning	781 [a]	10751 [a]	1115 [a]	516	125	62.5 [a]	1099.7	82.4 [a]	34.3	9.8
Previous best	1093	13519	1594	778	177	80.2	1500.4	100.6	43.2	13.4

Lower values are better. [a] best method for each horizon in each column; [b] score is significantly lower than the mean combination; [c] score is significantly lower than the median combination.
