# Supplementary material for: Interval forecasts of weekly incident and cumulative COVID-19 mortality in the United States: A comparison of combining methods
Source: PLoS One. 2022 Mar 29;17(3):e0266096. doi: 10.1371/journal.pone.0266096 (PMC8963571; doi:10.1371/journal.pone.0266096)
Supplement: S3 Table — (PDF) [file pone.0266096.s004.pdf]

S3 Table. For incident mortality, calibration for all locations.

Quantile	Mean	Median	Ensemble	Sym trim	Exterior trim	Interior trim	Envelope	Inv score	Inv score tuning	Previous best
1	5.2	2.8	2.8	3.7	5.8	2.9	1.4	3.1	3.3	4.1
2.5	7.0	4.3	4.2	5.3	7.6	3.8	1.4	4.8	4.7	5.6
5	8.9	6.0	5.8	6.9	9.7	5.3	1.5	6.9	6.6	7.6
10	12.9	9.5	9.0	10.2	14.1	8.0	1.5	10.8	10.1	12.2
15	17.0	13.0	12.5	13.8	18.2	11.6	1.6	15.0	14.3	16.9
20	20.8	16.6	16.0	17.1	22.2	15.1	1.7	19.1	18.4	20.9
25	24.9	20.6	19.9	20.9	26.4	18.7	1.8	23.1	22.8	24.9
30	29.0	24.7	24.0	25.0	30.9	22.6	1.9	27.4	27.0	29.1
35	33.7	29.2	28.5	29.3	36.0	27.3	2.1	32.0	31.6	33.2
40	38.6	33.6	33.0	34.1	41.4	32.6	2.5	36.5	36.0	36.7
45	43.4	38.2	37.9	39.0	47.0	38.4	3.0	41.4	41.0	40.9
50	49.2	43.6	44.1	44.5	48.1	44.4	3.9	46.9	46.4	44.7
55	56.0	49.7	51.3	50.9	49.9	58.1	95.7	54.0	52.6	49.0
60	61.4	54.8	56.3	56.3	56.0	63.7	96.5	59.4	57.6	52.6
65	66.6	59.4	60.7	61.3	61.9	69.2	97.2	64.6	62.7	56.9
70	71.8	63.9	65.1	66.2	67.3	74.3	97.9	69.9	67.6	61.4
75	76.3	68.5	69.7	70.8	72.1	79.0	98.4	74.7	72.5	65.6
80	81.2	73.0	74.2	75.8	77.4	83.4	98.8	79.8	77.7	70.4
80	85.5	78.2	79.3	80.8	81.8	87.6	99.1	84.7	82.8	74.9
90	89.7	83.0	83.8	85.9	86.4	91.4	99.4	89.5	87.4	79.7
95	93.7	88.4	89.1	90.9	90.8	95.1	99.6	94.0	92.6	85.9
97.5	95.9	91.5	92.1	93.8	93.4	97.0	99.7	96.4	95.4	89.6
99	97.5	93.9	94.3	95.5	95.4	98.3	99.8	97.9	97.1	92.3
