# Supplementary material for: Interval forecasts of weekly incident and cumulative COVID-19 mortality in the United States: A comparison of combining methods
Source: PLoS One. 2022 Mar 29;17(3):e0266096. doi: 10.1371/journal.pone.0266096 (PMC8963571; doi:10.1371/journal.pone.0266096)
Supplement: S4 Table — (PDF) [file pone.0266096.s005.pdf]

S4 Table. For incident mortality, calibration for U.S.

Quantile	Mean	Median	Ensemble	Sym trim	Exterior trim	Interior trim	Envelope	Inv score	Inv score tuning	Previous best
1	0.3	0.7	1.7	0.0	0.7	0.3	0.0	0.0	0.0	2.4
2.5	1.7	1.4	2.4	1.4	1.7	1.4	0.0	0.3	0.3	3.4
5	2.8	2.1	3.1	2.8	2.8	1.0	0.0	2.1	2.4	4.8
10	3.5	2.8	4.1	3.5	3.5	1.7	0.0	2.8	3.1	7.9
15	5.9	4.1	6.2	5.2	5.9	2.8	0.0	4.1	6.2	10.7
20	8.3	5.9	8.3	6.2	8.3	4.8	0.3	6.5	8.3	17.3
25	11.4	9.0	11.4	9.6	12.1	7.9	0.3	10.7	11.1	17.9
30	15.5	15.5	17.6	15.9	16.2	11.0	0.3	13.1	14.8	21.7
35	22.1	18.6	20.4	18.6	22.1	17.6	0.3	18.7	18.6	21.7
40	25.6	24.2	28.0	23.8	27.3	22.8	0.7	22.1	21.4	22.4
45	32.1	30.0	33.1	29.3	38.0	28.3	1.0	27.6	23.8	26.5
50	37.7	35.2	38.0	34.5	38.0	33.5	1.4	33.5	29.0	29.3
55	45.2	40.7	43.4	41.4	41.1	47.0	95.9	43.1	36.5	31.3
60	51.8	44.9	48.3	45.2	48.7	53.5	96.9	48.3	44.2	33.4
65	57.0	50.7	53.1	52.1	55.9	61.4	97.6	56.2	51.4	37.5
70	64.5	58.6	60.0	59.3	60.4	65.5	98.3	63.1	55.9	41.7
75	70.0	64.5	63.8	65.1	68.3	72.4	99.0	69.7	63.1	46.5
80	75.5	68.3	67.6	69.3	73.8	78.3	99.3	78.3	70.7	54.4
80	81.4	74.1	73.7	77.2	80.0	83.1	99.3	83.8	79.0	60.3
90	86.9	79.6	81.0	83.4	85.5	89.6	99.7	89.6	85.2	65.4
95	93.5	89.6	89.6	92.4	90.3	94.8	99.7	95.5	92.4	79.3
97.5	95.9	92.7	92.7	94.1	92.4	95.9	100.0	97.9	96.2	91.7
99	99.3	94.5	94.8	96.6	94.8	99.3	100.0	99.0	96.2	94.5
