# Supplementary material for: Interval forecasts of weekly incident and cumulative COVID-19 mortality in the United States: A comparison of combining methods
Source: PLoS One. 2022 Mar 29;17(3):e0266096. doi: 10.1371/journal.pone.0266096 (PMC8963571; doi:10.1371/journal.pone.0266096)
Supplement: S5 Table — (PDF) [file pone.0266096.s006.pdf]

S5 Table. For incident mortality, calibration for high mortality locations.

Quantile	Mean	Median	Ensemble	Sym trim	Exterior trim	Interior trim	Envelope	Inv score	Inv score tuning	Previous best
1	2.6	1.5	1.5	1.5	3.2	1.8	0.3	1.4	1.5	3.1
2.5	4.2	2.8	2.8	3.0	4.9	2.6	0.4	2.6	2.7	4.1
5	5.8	4.1	4.0	4.5	6.5	3.9	0.4	4.4	4.2	6.2
10	8.9	6.5	6.4	7.1	10.1	5.7	0.4	7.2	6.7	10.4
15	12.9	9.3	9.1	10.6	14.1	9.1	0.5	10.9	10.4	14.1
20	16.4	12.7	12.5	13.8	17.9	12.3	0.6	14.8	14.0	17.9
25	20.4	16.5	16.3	17.3	22.0	15.2	0.8	18.4	18.2	21.6
30	24.8	20.4	20.4	21.4	26.6	18.9	0.8	22.6	22.5	25.3
35	29.6	25.4	25.5	26.0	32.0	23.2	0.9	27.8	27.4	30.8
40	35.1	30.4	30.7	31.1	38.0	28.9	1.1	32.7	32.4	35.6
45	40.5	35.2	35.8	36.4	43.5	35.2	1.4	38.3	37.8	39.7
50	46.6	40.9	41.6	42.1	45.2	41.3	1.7	44.2	43.8	43.5
55	52.4	46.9	48.3	48.6	47.1	55.2	96.4	51.0	50.2	48.4
60	58.2	52.7	53.8	54.5	52.8	61.4	97.3	57.0	55.3	52.8
65	64.1	57.8	59.0	60.6	59.4	67.3	98.0	63.0	61.6	57.2
70	69.8	62.6	63.9	65.9	65.5	72.4	98.3	68.8	66.8	62.0
75	75.0	67.6	68.7	71.3	71.2	77.7	98.8	74.2	72.4	65.9
80	80.7	72.8	74.2	77.2	76.8	83.0	99.1	80.1	78.2	71.9
85	85.6	78.6	79.8	82.5	81.7	87.4	99.4	85.7	83.4	76.6
90	90.5	84.0	84.6	87.8	86.9	91.6	99.6	91.0	88.6	81.5
95	94.6	89.6	90.1	92.2	91.4	95.6	99.7	95.1	93.5	88.2
97.5	96.8	92.8	93.3	95.0	94.4	97.4	99.8	97.4	96.3	91.5
99	98.2	95.1	95.4	96.4	96.0	98.4	99.9	98.6	97.7	93.4
