# Supplementary material for: Interval forecasts of weekly incident and cumulative COVID-19 mortality in the United States: A comparison of combining methods
Source: PLoS One. 2022 Mar 29;17(3):e0266096. doi: 10.1371/journal.pone.0266096 (PMC8963571; doi:10.1371/journal.pone.0266096)
Supplement: S6 Table — (PDF) [file pone.0266096.s007.pdf]

S6 Table. For incident mortality, calibration for medium mortality locations.

Quantile	Mean	Median	Ensemble	Sym trim	Exterior trim	Interior trim	Envelope	Inv score	Inv score tuning	Previous best
1	3.7	1.8	1.8	2.5	4.4	1.7	0.5	2.0	2.1	3.1
2.5	5.6	2.9	2.9	4.1	6.1	2.7	0.5	3.4	3.6	4.9
5	7.4	4.6	4.5	5.6	8.2	4.0	0.5	5.4	5.5	6.6
10	11.7	8.1	7.9	9.0	13.0	6.9	0.6	9.6	9.1	11.4
15	15.7	11.4	11.4	12.9	16.9	10.5	0.6	14.0	13.4	17.0
20	19.7	15.2	14.9	16.1	21.0	13.8	0.7	18.1	17.9	21.0
25	24.1	19.2	18.8	20.0	25.4	17.8	0.8	22.5	22.5	25.1
30	28.5	23.7	23.2	24.4	30.7	21.7	0.9	27.1	27.0	29.8
35	33.9	28.3	28.0	29.2	36.4	26.8	1.2	32.4	32.1	33.5
40	39.1	33.0	32.7	34.1	42.3	32.6	1.5	37.2	36.8	36.5
45	44.1	38.4	38.3	39.8	49.6	38.8	1.9	42.7	42.4	40.7
50	50.9	44.6	45.1	45.9	50.7	45.5	2.6	48.9	48.4	44.5
55	57.4	51.1	52.5	52.8	52.0	59.2	96.0	55.8	54.9	49.4
60	62.6	56.5	57.6	58.5	57.8	64.7	96.5	61.0	60.2	53.8
65	67.6	61.2	62.3	63.3	63.6	69.9	97.1	66.1	65.1	59.1
70	72.4	65.5	66.7	67.8	69.1	74.9	97.9	71.2	69.8	63.5
75	76.3	70.5	71.6	72.5	73.3	79.3	98.2	75.6	74.4	68.1
80	81.3	74.5	75.7	77.3	78.3	83.4	98.7	80.8	79.9	73.1
85	85.7	79.4	80.4	82.5	83.0	87.6	99.0	85.4	85.0	77.3
90	89.9	84.2	84.9	86.9	87.4	91.4	99.3	90.2	89.1	81.4
95	94.1	89.2	89.7	91.4	91.6	95.2	99.6	94.3	93.7	87.2
97.5	96.2	91.9	92.5	94.0	93.9	96.9	99.7	96.6	96.3	90.3
99	97.6	94.5	94.9	95.8	95.9	98.1	99.7	97.9	97.0	92.7
