# Supplementary material for: Interval forecasts of weekly incident and cumulative COVID-19 mortality in the United States: A comparison of combining methods
Source: PLoS One. 2022 Mar 29;17(3):e0266096. doi: 10.1371/journal.pone.0266096 (PMC8963571; doi:10.1371/journal.pone.0266096)
Supplement: S7 Table — (PDF) [file pone.0266096.s008.pdf]

S7 Table. For incident mortality, calibration for low mortality locations.

Quantile	Mean	Median	Ensemble	Sym trim	Exterior trim	Interior trim	Envelope	Inv score	Inv score tuning	Previous best
1	9.2	5.9	5.8	7.1	10.0	6.1	3.8	6.4	6.2	6.3
2.5	11.2	8.0	7.7	8.7	12.1	7.1	3.8	8.5	7.8	8.0
5	13.3	10.0	9.5	10.7	14.7	9.1	3.8	11.2	10.3	10.4
10	18.4	14.9	13.9	14.7	19.4	12.6	3.8	16.0	14.9	15.8
15	22.9	19.7	18.1	18.4	24.1	16.3	3.8	20.6	19.9	20.4
20	27.1	23.6	22.3	22.9	28.8	20.8	4.0	25.4	24.4	24.2
25	31.7	28.6	26.6	27.1	33.2	24.8	4.2	29.7	28.7	28.6
30	35.6	32.9	31.0	31.3	37.2	28.9	4.4	34.2	32.8	32.8
35	39.7	37.1	35.0	35.4	41.7	33.9	4.7	38.2	37.0	36.6
40	44.1	41.3	38.8	40.2	46.6	38.8	5.3	42.4	41.2	40.1
45	48.4	45.1	42.9	44.1	51.0	44.1	6.2	46.5	45.5	44.5
50	53.2	49.6	49.7	49.1	52.0	49.8	8.1	51.2	50.2	48.3
55	61.5	55.7	57.6	55.6	54.3	63.8	96.5	59.1	56.1	52.4
60	67.1	60.1	62.2	60.5	61.7	68.6	97.3	64.6	61.1	54.7
65	71.9	64.1	65.8	64.7	67.2	74.0	98.0	68.9	65.4	58.6
70	76.8	68.0	69.5	69.3	71.7	79.0	98.6	73.3	70.1	63.3
75	80.8	71.9	73.4	72.8	75.8	82.8	99.2	77.5	74.4	67.9
80	85.3	75.8	77.3	77.1	80.8	87.2	99.5	81.8	78.8	72.0
85	89.0	80.7	81.9	81.4	84.0	90.6	99.7	86.4	83.5	76.3
90	92.1	85.1	85.8	86.6	88.0	93.5	99.8	90.8	87.9	81.7
95	95.4	90.1	90.7	91.9	92.0	96.3	99.9	95.0	93.1	87.0
97.5	97.3	93.0	93.6	94.7	94.4	98.0	100.0	97.0	95.7	90.7
99	98.7	94.9	95.3	96.3	96.6	99.1	100.0	98.8	97.6	93.0
