# Supplementary material for: Interval forecasts of weekly incident and cumulative COVID-19 mortality in the United States: A comparison of combining methods
Source: PLoS One. 2022 Mar 29;17(3):e0266096. doi: 10.1371/journal.pone.0266096 (PMC8963571; doi:10.1371/journal.pone.0266096)
Supplement: S8 Table — Lower values are better. a best method for each horizon in each column; b score is significantly lower than the mean combination; c score is significantly lower than the median combination. (PDF) [file pone.0266096.s009.pdf]

S8 Table. For cumulative mortality, 95% interval MIS and MWIS for each prediction horizon.

Method	95% interval MIS					MWIS				
	All	U.S.	High	Med	Low	All	U.S.	High	Med	Low
1 week ahead										
Mean	6283	107370	9357	2445	1101	202	3806	283	78	34
Median	2355 [ab]	29080 [b]	4791 [b]	465 [ab]	237 [b]	93 [ab]	1567 [b]	158 [b]	24 [ab]	11 [ab]
Ensemble	2364 [b]	30211 [b]	4749 [bc]	470 [b]	235 [ab]	93 [ab]	1559 [ab]	157 [abc]	24 [ab]	11 [ab]
Sym trim	2723 [b]	32310 [b]	5376 [b]	694 [b]	359 [b]	101 [b]	1652 [b]	171 [b]	28 [b]	13 [b]
Exterior trim	5642 [b]	92082 [b]	8593 [b]	2205 [b]	1043 [b]	166 [b]	2893 [b]	246 [b]	63 [b]	29 [b]
Interior trim	2444 [b]	23852	4379 [ab]	1075 [b]	619 [b]	134 [b]	2120 [b]	206 [b]	52 [b]	26 [b]
Envelope	5076 [b]	97199	7209 [b]	1941 [b]	659 [b]	780	17437	927	329	103
Inv score	3737 [b]	30588 [b]	6840 [b]	1878 [b]	914 [b]	130 [b]	1865 [b]	208 [b]	54 [b]	27 [b]
Inv score tuning	4225 [b]	22738 [b]	7784	2961	842	128 [b]	1644 [b]	214 [b]	57 [b]	24 [b]
Previous best	5232	20629 [ab]	9196	5120	473	130 [b]	1609 [b]	218 [b]	69	17 [b]
2 weeks ahead										
Mean	5912	89926	9250	2463	1080	227	3907	335	92	37
Median	2574 [ab]	26967 [b]	5222 [b]	788 [ab]	278 [ab]	123 [ab]	1966 [b]	201 [b]	43 [ab]	16 [ab]
Ensemble	2630 [b]	30649 [b]	5164 [ab]	800 [b]	279 [b]	124 [b]	2038 [b]	199 [ab]	43 [ab]	16 [ab]
Sym trim	2894 [b]	28487 [b]	5792 [b]	1000 [b]	384 [b]	131 [b]	1994 [b]	218 [b]	47 [b]	18 [b]
Exterior trim	5687 [b]	85822	8922 [b]	2370 [b]	1056	196 [b]	3127 [b]	303 [b]	80 [b]	33 [b]
Interior trim	3157 [b]	25998 [b]	6126	1334 [b]	667 [b]	208	2518 [b]	387	71 [b]	31 [b]
Envelope	6525	114668	9762	2510	942	954	19297	1278	384	121
Inv score	3785 [b]	24350 [b]	7121 [b]	2088	937	162 [b]	2135 [b]	266 [b]	71 [b]	32 [b]
Inv score tuning	4301 [b]	19548 [ab]	7940	3198	869	154 [b]	1933 [ab]	254 [b]	75 [b]	29 [b]
Previous best	5684	27102 [b]	9635	5597	559	166 [b]	2100 [b]	266 [b]	95	23
3 weeks ahead										
Mean	4971	78958	8254	1800	507	235	4203	355	89	27
Median	2873 [b]	29160	5671 [b]	1077 [b]	326 [b]	156 [b]	2488 [b]	247 [b]	63 [ab]	20 [ab]
Ensemble	2907 [b]	31733	5617 [b]	1082 [b]	328 [b]	155 [ab]	2484 [b]	245 [ab]	64 [b]	20 [ab]
Sym trim	3040 [b]	34160	5832 [b]	1138 [b]	320 [b]	162 [b]	2591 [b]	258 [b]	65 [b]	20 [ab]
Exterior trim	5034	80925	8288	1843	508	212 [b]	3552 [b]	332 [b]	81 [b]	25 [b]
Interior trim	3293	31080	6874	1069 [ab]	301 [b]	252	3136	491	73 [b]	23 [b]
Envelope	8114	147130	11750	3032	1381	1081	21951	1499	403	113
Inv score	2908 [b]	27823	5911 [b]	1070 [b]	277 [ab]	172 [b]	2627 [b]	285 [b]	67 [b]	21 [b]
Inv score tuning	2791 [ab]	24017 [ab]	5604 [ab]	1231 [b]	290 [b]	159 [b]	2385 [ab]	257 [b]	69 [b]	21 [b]
Previous best	3764	36374	7336	1664	373 [b]	183 [b]	2793	280	90	24
4 weeks ahead										
Mean	4993	73035	8425	1985	568	271	4706	410	109	32
Median	3335 [b]	35545	6373 [b]	1352 [ab]	384 [b]	200 [ab]	3306	307 [b]	85 [ab]	26 [ab]
Ensemble	3384 [b]	39995	6248 [b]	1368 [b]	383 [b]	200 [ab]	3338	302 [ab]	86 [b]	26 [ab]
Sym trim	3517 [b]	41194	6527 [b]	1435 [b]	375 [b]	208 [b]	3423	320 [b]	88 [b]	26 [ab]
Exterior trim	5188	76633	8726	2092	544	254 [b]	4195	397	103 [b]	30 [b]
Interior trim	3920	40403	7794	1403 [b]	417 [b]	311	3960	592	96 [b]	30 [b]
Envelope	11944	258403	14536	4241	2558	1292	25886	1789	490	151
Inv score	3286 [b]	33773	6369 [b]	1356 [b]	340 [ab]	216 [b]	3355	346 [b]	89 [b]	26 [ab]
Inv score tuning	3187 [ab]	32193 [a]	5943 [ab]	1558 [b]	354 [b]	201 [b]	3117 [a]	312 [b]	92 [b]	27 [b]
Previous best	4465	48258	8206	2146	466	240	3820	358	121	32

Lower values are better. [a] best method for each horizon in each column; [b] score is significantly lower than the mean combination; [c] score is significantly lower than the median combination.
