# Supplementary material for: Interval forecasts of weekly incident and cumulative COVID-19 mortality in the United States: A comparison of combining methods
Source: PLoS One. 2022 Mar 29;17(3):e0266096. doi: 10.1371/journal.pone.0266096 (PMC8963571; doi:10.1371/journal.pone.0266096)
Supplement: S9 Table — (PDF) [file pone.0266096.s010.pdf]

**S9 Table. For cumulative mortality, calibration for all locations.**

| Quantile | Mean | Median | Ensemble | Sym trim | Exterior trim | Interior trim | Envelope | Inv score | Inv score tuning | Previous best |
|---|---|---|---|---|---|---|---|---|---|---|
| *1* | 18.0 | 3.2 | 3.3 | 4.8 | 19.1 | 3.1 | 1.3 | 5.1 | 4.6 | 4.4 |
| *2.5* | 20.0 | 4.5 | 4.6 | 6.1 | 21.2 | 4.2 | 1.4 | 7.3 | 6.3 | 6.2 |
| *5* | 22.1 | 6.2 | 6.3 | 7.8 | 23.7 | 5.8 | 1.5 | 10.1 | 8.5 | 9.0 |
| *10* | 26.0 | 9.6 | 9.6 | 11.0 | 28.2 | 8.9 | 1.6 | 14.9 | 12.0 | 13.8 |
| *15* | 29.9 | 12.9 | 13.1 | 14.5 | 32.3 | 12.1 | 1.7 | 19.5 | 16.3 | 18.0 |
| *20* | 33.6 | 16.6 | 16.7 | 17.8 | 36.2 | 15.5 | 1.7 | 23.5 | 20.1 | 21.7 |
| *25* | 37.2 | 20.2 | 20.6 | 21.3 | 39.9 | 19.5 | 1.9 | 27.6 | 24.4 | 25.8 |
| *30* | 41.1 | 24.3 | 24.8 | 25.7 | 44.0 | 23.6 | 2.0 | 32.0 | 28.9 | 30.2 |
| *35* | 44.9 | 29.1 | 29.4 | 30.2 | 48.2 | 27.9 | 2.3 | 36.2 | 33.4 | 34.2 |
| *40* | 48.9 | 33.5 | 33.8 | 34.7 | 52.6 | 32.7 | 2.5 | 40.8 | 38.1 | 38.0 |
| *45* | 53.1 | 38.1 | 38.4 | 39.4 | 55.6 | 38.7 | 3.1 | 45.5 | 43.2 | 42.0 |
| *50* | 58.0 | 43.7 | 44.8 | 45.6 | 56.5 | 45.1 | 4.0 | 51.3 | 48.9 | 46.1 |
| *55* | 63.5 | 50.4 | 52.2 | 52.5 | 57.5 | 66.0 | 95.0 | 58.0 | 55.3 | 50.9 |
| *60* | 67.9 | 55.7 | 57.4 | 58.0 | 60.9 | 70.5 | 95.7 | 63.0 | 60.5 | 55.3 |
| *65* | 71.8 | 60.3 | 61.8 | 62.7 | 65.6 | 74.1 | 96.3 | 67.6 | 64.9 | 59.0 |
| *70* | 75.4 | 64.7 | 66.1 | 67.1 | 69.4 | 77.7 | 96.8 | 71.8 | 69.6 | 62.9 |
| *75* | 78.9 | 69.0 | 70.4 | 71.4 | 73.4 | 81.2 | 97.3 | 75.6 | 73.9 | 67.0 |
| *80* | 82.1 | 73.5 | 74.7 | 75.7 | 76.7 | 84.5 | 97.7 | 79.7 | 78.1 | 71.0 |
| *85* | 85.4 | 77.9 | 78.9 | 80.2 | 80.4 | 87.9 | 98.1 | 83.8 | 82.5 | 75.7 |
| *90* | 88.6 | 82.3 | 83.0 | 84.8 | 84.0 | 90.9 | 98.4 | 87.6 | 86.7 | 80.1 |
| *95* | 92.1 | 87.0 | 87.6 | 89.1 | 88.6 | 94.3 | 98.8 | 91.8 | 90.8 | 85.5 |
| *97.5* | 94.1 | 90.1 | 90.4 | 91.8 | 91.3 | 96.2 | 99.1 | 94.4 | 93.3 | 88.9 |
| *99* | 95.8 | 92.3 | 92.5 | 93.9 | 93.6 | 97.6 | 99.3 | 96.1 | 95.3 | 91.7 |
