# Supplementary material for: Interval forecasts of weekly incident and cumulative COVID-19 mortality in the United States: A comparison of combining methods
Source: PLoS One. 2022 Mar 29;17(3):e0266096. doi: 10.1371/journal.pone.0266096 (PMC8963571; doi:10.1371/journal.pone.0266096)
Supplement: S10 Table — (PDF) [file pone.0266096.s011.pdf]

S10 Table. For cumulative mortality, calibration for U.S.

Quantile	Mean	Median	Ensemble	Sym trim	Exterior trim	Interior trim	Envelope	Inv score	Inv score tuning	Previous best
1	16.0	0.0	0.6	1.3	16.3	0.0	0.0	1.6	0.0	0.0
2.5	17.0	0.0	0.6	1.3	17.3	0.0	0.0	2.9	0.3	0.0
5	18.3	0.0	0.6	1.6	18.6	0.0	0.0	5.5	1.0	1.9
10	18.9	1.6	1.9	3.6	19.9	0.0	0.0	7.5	2.6	10.1
15	21.9	3.6	4.9	6.5	22.2	2.3	0.0	10.4	5.2	11.7
20	22.5	6.8	7.1	7.8	24.2	4.5	0.0	12.4	8.8	14.3
25	25.8	8.8	9.4	9.4	25.8	6.2	0.0	13.7	12.7	18.3
30	27.8	13.0	14.3	12.4	28.1	10.4	0.0	18.3	15.0	22.5
35	30.4	16.0	17.6	17.6	32.4	15.0	0.0	21.2	17.6	23.8
40	34.7	19.6	20.5	19.3	35.3	19.6	0.0	23.8	20.2	26.4
45	37.9	23.8	24.5	25.2	38.9	24.5	0.6	27.4	24.8	32.3
50	43.5	29.4	30.4	32.4	41.5	30.1	1.0	31.4	28.8	38.5
55	48.1	35.0	36.0	37.0	43.2	48.4	91.9	38.3	35.9	43.8
60	51.0	38.9	40.2	40.6	45.2	51.7	92.5	43.9	43.9	46.4
65	54.3	41.2	43.5	42.5	49.4	55.0	93.2	47.4	47.1	47.1
70	58.3	44.9	45.2	46.8	53.1	58.3	93.8	51.4	52.7	50.0
75	61.2	51.4	51.1	52.7	56.0	63.2	95.5	58.0	56.0	54.0
80	64.8	57.3	56.9	57.9	59.9	67.8	96.8	63.5	60.9	60.2
85	71.7	62.9	62.2	66.1	66.8	76.2	97.7	70.1	68.4	65.7
90	76.9	69.1	69.1	75.6	74.3	81.5	99.0	77.9	77.3	71.6
95	84.7	79.5	78.5	84.4	82.1	87.3	99.7	85.1	88.0	89.9
97.5	89.3	84.4	82.4	87.0	85.7	91.9	100.0	91.6	92.9	89.3
99	92.3	87.7	85.4	89.6	89.0	94.5	100.0	95.2	95.5	95.5
