# Supplementary material for: Interval forecasts of weekly incident and cumulative COVID-19 mortality in the United States: A comparison of combining methods
Source: PLoS One. 2022 Mar 29;17(3):e0266096. doi: 10.1371/journal.pone.0266096 (PMC8963571; doi:10.1371/journal.pone.0266096)
Supplement: S11 Table — (PDF) [file pone.0266096.s012.pdf]

S11 Table. For cumulative mortality, calibration for high mortality locations.

Quantile	Mean	Median	Ensemble	Sym trim	Exterior trim	Interior trim	Envelope	Inv score	Inv score tuning	Previous best
1	19.0	4.8	4.9	5.6	20.3	4.4	2.3	5.5	5.5	5.1
2.5	21.1	6.0	6.0	6.8	22.6	5.2	2.3	7.5	7.0	6.9
5	23.1	7.6	7.6	8.0	24.9	6.7	2.4	9.8	8.4	9.5
10	26.8	10.4	10.4	11.0	29.1	9.6	2.5	14.3	11.2	13.5
15	30.4	13.0	13.3	14.0	32.8	12.8	2.5	18.4	14.5	17.9
20	33.8	16.5	16.6	17.6	36.5	15.7	2.7	22.4	17.5	22.1
25	37.4	20.4	21.0	21.0	40.4	19.3	3.0	26.4	21.9	26.2
30	41.0	24.5	25.4	25.7	44.3	23.1	3.2	30.6	26.5	30.5
35	44.6	29.7	30.4	30.2	48.1	27.8	3.3	35.0	31.1	35.2
40	48.8	33.9	35.2	34.8	52.7	32.5	3.5	40.0	36.3	39.9
45	53.2	38.9	40.2	40.0	56.0	39.6	3.8	45.5	42.8	43.9
50	58.4	44.6	46.3	46.2	57.2	45.7	4.1	51.4	49.2	48.5
55	63.3	51.3	53.3	52.2	58.0	66.6	97.0	58.1	56.1	55.1
60	68.4	57.3	59.2	58.7	61.7	71.6	97.8	63.8	62.3	60.3
65	72.7	62.3	64.2	64.3	66.5	75.2	98.2	69.0	67.0	64.1
70	76.6	67.5	69.1	69.3	70.7	78.8	98.6	73.4	72.3	68.0
75	80.6	72.2	73.7	74.3	75.3	82.7	98.9	78.2	77.3	72.2
80	83.8	76.7	78.0	78.2	78.5	86.2	99.0	82.3	81.5	76.6
85	87.4	81.1	82.3	83.1	82.1	90.1	99.3	86.4	86.0	80.8
90	90.9	85.6	86.6	87.8	85.6	93.0	99.5	90.4	89.2	86.0
95	94.1	90.3	91.1	92.1	90.9	95.8	99.6	94.3	93.2	89.9
97.5	95.9	93.5	93.9	94.5	93.1	97.4	99.7	96.9	95.5	92.9
99	97.4	95.7	95.7	96.4	95.2	98.4	99.8	98.3	97.6	94.4
