# Supplementary material for: Interval forecasts of weekly incident and cumulative COVID-19 mortality in the United States: A comparison of combining methods
Source: PLoS One. 2022 Mar 29;17(3):e0266096. doi: 10.1371/journal.pone.0266096 (PMC8963571; doi:10.1371/journal.pone.0266096)
Supplement: S12 Table — (PDF) [file pone.0266096.s013.pdf]

S12 Table. For cumulative mortality, calibration for medium mortality locations.

Quantile	Mean	Median	Ensemble	Sym trim	Exterior trim	Interior trim	Envelope	Inv score	Inv score tuning	Previous best
1	17.7	1.5	1.6	2.7	18.9	1.8	0.2	3.6	3.1	3.3
2.5	19.8	2.7	2.8	4.2	21.1	2.8	0.3	6.0	5.0	4.9
5	22.0	4.2	4.4	5.8	23.8	4.0	0.4	8.9	7.3	8.2
10	26.0	7.6	7.7	9.1	28.2	7.2	0.4	13.7	10.6	13.6
15	29.9	10.5	10.7	12.5	32.6	10.5	0.4	18.4	15.5	17.3
20	33.8	14.1	14.4	15.6	36.8	14.4	0.5	22.9	19.4	20.8
25	37.6	18.0	18.2	19.3	40.6	18.7	0.5	27.3	23.9	24.9
30	42.2	22.6	23.1	24.6	44.8	23.1	0.6	32.3	28.8	29.8
35	46.7	27.8	28.3	29.4	49.8	27.3	0.9	36.9	34.2	33.9
40	50.6	32.7	33.2	34.4	54.1	32.6	1.1	41.8	39.4	37.4
45	54.9	37.7	38.1	39.4	57.3	38.5	1.6	46.0	44.3	42.5
50	60.0	44.3	45.2	46.3	58.0	45.6	2.2	52.7	50.8	47.1
55	65.4	52.0	53.3	54.2	58.9	68.0	96.5	59.2	57.1	51.9
60	69.8	57.6	58.8	59.8	62.8	72.3	97.1	64.5	62.7	56.2
65	73.6	62.4	63.2	64.8	67.5	76.0	97.5	69.0	67.5	60.4
70	77.2	66.8	67.5	69.2	71.4	79.6	98.0	73.5	72.3	65.1
75	80.7	70.7	71.6	73.2	75.3	82.7	98.5	77.1	76.7	69.6
80	83.8	75.3	76.2	77.8	78.5	86.1	98.9	81.4	80.9	73.3
85	87.4	80.1	80.9	82.2	82.4	89.3	99.2	85.9	85.6	77.6
90	90.6	84.9	85.6	86.8	86.2	92.1	99.4	89.7	89.6	81.6
95	94.2	89.4	89.8	91.0	90.5	95.5	99.6	94.0	93.0	86.9
97.5	96.1	92.6	92.9	94.0	93.3	97.0	99.7	96.1	95.4	90.4
99	97.5	94.7	94.9	96.0	95.6	98.1	99.9	97.6	97.0	93.0
