# Supplementary material for: Interval forecasts of weekly incident and cumulative COVID-19 mortality in the United States: A comparison of combining methods
Source: PLoS One. 2022 Mar 29;17(3):e0266096. doi: 10.1371/journal.pone.0266096 (PMC8963571; doi:10.1371/journal.pone.0266096)
Supplement: S13 Table — (PDF) [file pone.0266096.s014.pdf]

S13 Table. For cumulative mortality, calibration for low mortality locations

Quantile	Mean	Median	Ensemble	Sym trim	Exterior trim	Interior trim	Envelope	Inv score	Inv score tuning	Previous best
1	17.0	3.5	3.5	4.4	18.4	3.9	1.5	4.7	4.6	5.4
2.5	19.3	5.4	5.4	6.0	20.7	5.6	1.8	7.1	6.2	7.1
5	21.7	7.6	7.5	8.5	23.9	7.9	1.9	10.8	9.4	10.1
10	26.7	11.8	11.8	12.7	29.8	12.1	2.0	16.1	14.3	15.3
15	31.2	16.6	16.4	17.1	34.6	15.8	2.0	21.6	19.3	20.2
20	35.6	21.1	20.8	21.4	39.0	20.0	2.1	26.0	24.9	24.1
25	39.5	25.3	25.3	25.2	43.2	24.7	2.2	30.7	28.9	29.0
30	43.6	29.4	29.4	29.2	48.2	29.2	2.3	35.1	33.7	33.5
35	47.6	34.5	33.9	33.9	52.6	33.0	2.7	39.6	37.8	37.5
40	52.1	39.0	37.9	38.6	57.0	37.8	3.1	44.4	42.1	41.5
45	56.9	43.5	42.5	43.5	60.3	43.5	4.2	49.7	47.3	44.7
50	62.2	48.9	50.0	50.0	60.4	50.2	5.8	55.3	52.8	48.4
55	68.9	55.3	58.0	57.2	61.6	71.0	97.4	63.4	59.7	52.1
60	73.4	60.1	62.7	62.4	64.8	75.3	98.1	67.8	64.3	56.7
65	77.4	64.6	66.7	66.5	69.8	79.1	98.7	72.4	68.5	60.3
70	81.6	69.0	71.1	71.0	73.5	83.0	99.1	76.6	72.8	63.8
75	84.8	73.7	75.8	74.9	77.7	86.3	99.3	80.4	77.0	68.1
80	88.2	78.4	79.8	79.5	81.5	89.4	99.6	84.4	81.2	72.0
85	90.8	82.6	83.9	84.3	85.1	92.0	99.7	88.4	85.5	77.6
90	93.5	86.7	87.3	88.4	88.2	94.7	99.9	91.5	89.8	81.9
95	96.5	91.2	92.0	92.4	92.7	97.2	100.0	95.1	93.7	87.0
97.5	98.0	94.0	94.2	94.8	95.0	98.4	100.0	97.4	95.9	91.1
99	99.1	96.0	96.2	96.8	96.7	99.3	100.0	98.8	97.4	93.3
