# Supplementary material for: Interval forecasts of weekly incident and cumulative COVID-19 mortality in the United States: A comparison of combining methods
Source: PLoS One. 2022 Mar 29;17(3):e0266096. doi: 10.1371/journal.pone.0266096 (PMC8963571; doi:10.1371/journal.pone.0266096)
Supplement: S14 Table — Shows percentages. Higher values are better. a best method in each column. (PDF) [file pone.0266096.s015.pdf]

S14 Table. Sensitivity analysis for incident mortality, skill scores of the 95% interval MIS and MWIS after excluding locations for which there were noticeable changes in reporting patterns.

	95% interval MIS					MWIS				
**Method**	**All**	**U.S.**	**High**	**Med**	**Low**	**All**	**U.S.**	**High**	**Med**	**Low**
Mean	0.0	0.0	0.0	0.0	0.0	0.0	0.0	0.0	0.0	0.0
Median	9.9	-4.0	16.8	-1.8	16.3	6.0	-1.9	8.5	3.0	7.5
Ensemble	11.2	-11.4	18.7 [a]	-0.1	17.0 [a]	6.4 [a]	-3.1	9.4 [a]	3.4 [a]	7.5 [a]
Sym trim	9.4	-2.3	15.2	1.3	13.1	4.4	-1.7	4.9	2.3	6.8
Exterior trim	-2.3	-12.8	-3.3	-5.0	2.7	0.5	-3.1	0.5	-1.5	3.1
Interior trim	4.3	-0.5	3.0	5.1	4.9	0.5	-1.2	0.1	1.2	0.3
Envelope	-239.0	-503.8	-253.8	-168.2	-313.1	-258.5	-266.6	-297.2	-220.8	-267.4
Inv score	12.5 [a]	3.1	17.2	6.4 [a]	15.2	4.4	6.0	6.9	2.7	3.7
Inv score tuning	8.6	6.7 [a]	16.5	0.2	9.8	1.6	7.1 [a]	6.1	-1.5	-0.1
Previous best	-20.8	-23.5	-12.4	-35.2	-13.5	-22.7	-18.3	-13.8	-25.1	-30.3

Shows percentages. Higher values are better. [a] best method in each column.
