# Supplementary material for: Interval forecasts of weekly incident and cumulative COVID-19 mortality in the United States: A comparison of combining methods
Source: PLoS One. 2022 Mar 29;17(3):e0266096. doi: 10.1371/journal.pone.0266096 (PMC8963571; doi:10.1371/journal.pone.0266096)
Supplement: S15 Table — Shows percentages. Higher values are better. a best method in each column. (PDF) [file pone.0266096.s016.pdf]

S15 Table. Sensitivity analysis for cumulative mortality, skill scores of the 95% interval MIS and MWIS after excluding locations for which there were noticeable changes in reporting patterns.

	95% interval MIS					MWIS				
**Method**	**All**	**U.S.**	**High**	**Med**	**Low**	**All**	**U.S.**	**High**	**Med**	**Low**
Mean	0.0	0.0	0.0	0.0	0.0	0.0	0.0	0.0	0.0	0.0
Median	69.7 [a]	65.4	66.8 [a]	64.9 [a]	77.4 [a]	46.2 [a]	43.9	43.2	42.4	53.5 [a]
Ensemble	69.4	62.0	66.5	64.7	77.1	46.1	43.3	43.5 [a]	42.3 [a]	52.9
Sym trim	65.4	61.0	61.2	60.5	74.2	43.1	41.9	40.4	38.6	50.8
Exterior trim	5.7	4.0	5.4	3.1	9.2	12.5	17.2	12.1	10.2	15.2
Interior trim	47.4	65.3	49.6	47.9	42.6	18.9	29.4	21.3	19.7	14.4
Envelope	-56.4	-76.8	-47.9	-49.3	-74.4	-346.5	-408.8	-349.7	-356.5	-327.0
Inv score	34.0	66.6	41.7	28.5	27.7	24.9	39.9	29.6	23.1	20.3
Inv score tuning	22.4	71.8 [a]	28.9	2.8	30.0	24.4	45.4 [a]	28.8	20.4	22.4
Previous best	27.5	62.1	23.0	-15.9	59.9	19.4	37.9	17.8	7.4	32.1

Shows percentages. Higher values are better. [a] best method in each column.
